# Buccal mucosa for use in urethral reconstruction: evolution of use over the last 30 years

**DOI:** 10.3389/fruro.2023.1138707

**Published:** 2023-05-02

**Authors:** Jordan Foreman, Andrew Peterson, Kevin Krughoff

**Affiliations:** ^1^ Department of Surgery, Division of Urology, Duke University Health System, Durham, NC, United States; ^2^ Department of Urology, Oregon Urology Institute, Springfield, OR, United States

**Keywords:** urethroplasty, buccal mucosa, graft, complications, history

## Introduction and review of history

Over the course of urological history, there have been many different surgical techniques to treat urethral stricture disease. The basis of treatment has focused on procedures that offered a durable outcome, limited morbidity, and limited sexual side effects. Early management of urethral stricture disease revolved around the use of local flaps of penile and scrotal skin, with rates of failure around 20%–30% (1). A need for more durable outcomes resulted in an exploration of free graft substitution. Iterations included meshed split-thickness skin grafts (STSGs), with success rates of 80%, which require multiple stages and have morbidity associated with harvest (2). There is also bladder mucosa, with rates of failure around 12% at 28 months and morbidity associated with harvesting the graft with open surgery (3). The first described use of buccal mucosal grafts (BMGs) for urethral reconstruction was in the early 19th century by Sapezhko, and the buccal mucosa was characterized as the ideal graft tissue because of its robust epithelium, resistance to infection, and ease of transfer (1, 4). Interestingly, the use of free oral grafts for urethral stricture disease predated the use of STSGs and bladder mucosa but fell out of favor. The use of free oral grafts dates back to the early 1890s (4). It was not revived until 1941 when Humby first used BMGs for urethral reconstructions (5). Fast forward to 1996, and Morey and McAninch described a two-team approach and the use of BMGs for urethroplasty using a ventral onlay approach (6). In 1998, Barbagli et al. popularized a dorsal onlay approach using BMGs for bulbar urethral strictures, and in 2009 Kulkarni et al. described a unilateral dorsal onlay using BMGs (7, 8). At this present time, the use of buccal mucosa is the standard graft for substitution urethroplasty (9).

## Advantages of BMG

One fundamental technique in reconstructive urology is tissue transfer. To have an effective graft tissue there needs to be a wide availability of tissue and minimum harvest site morbidity, the graft must take to a vascular bed, and there needs to be ease of replication and harvest. A BMG is an ideal graft as the epithelium is thick with high elastic fiber content, the lamina propria is thin, and there is a wide availability with ease of harvest and minimal morbidity (10, 11).

Buccal mucosa is a non-keratinizing stratified squamous epithelium phenotypically similar to the penile and glandular urethra (12). It is exposed to a moist environment with natural immunity factors that protect the tissue from infection (13). The vascular characteristics of a BMG, which allow it to be an optimal graft for urethroplasty, are secondary to a panlaminar plexus, where the vascular supply penetrates from the submucosa to lamina propria (10, 14). This promotes angiogenesis and revascularization at the graft bed during graft take (10). Furthermore, when the lamina propria is harvested with epithelium, the graft can be thinned without altering its vascular or physical characteristics (14).

When compared with other substitution grafts for urethroplasty (lingual and lower lip), BMGs have fewer donor site complications (9, 11, 15–18). However, there are no reported differences in success rates of urethroplasty between BMGs and lingual grafts (11, 16, 17). Recently, both the American Urological Association (AUA) and the European Urological Association (EUA) guidelines promoted the preferential use of BMGs for urethral reconstruction over penile skin flaps (19, 20).

## Technical considerations during the harvest of BMGs

The buccal mucosa is innervated by the long buccal nerve of cranial nerve III and the superior alveolar nerves of cranial nerve II (12). The vascular supply stems from the buccal artery, which branches from the maxillary artery (12). The borders of the buccal mucosa include the vermilion border anteriorly, the retromolar trigone posteriorly, and the mandibular and maxillary mucolabial folds superiorly and inferiorly. Just lateral to the buccal mucosal and lamina propria is the buccinator muscle, which should be left intact to limit postoperative pain and speech and mastication difficulty. The most important anatomical landmark recognized at time of harvest is the parotid or Stensen’s duct. This is identified as a small raised nodule located on the mucosa adjacent to the maxillary second molar (12).

**Figure 1:**
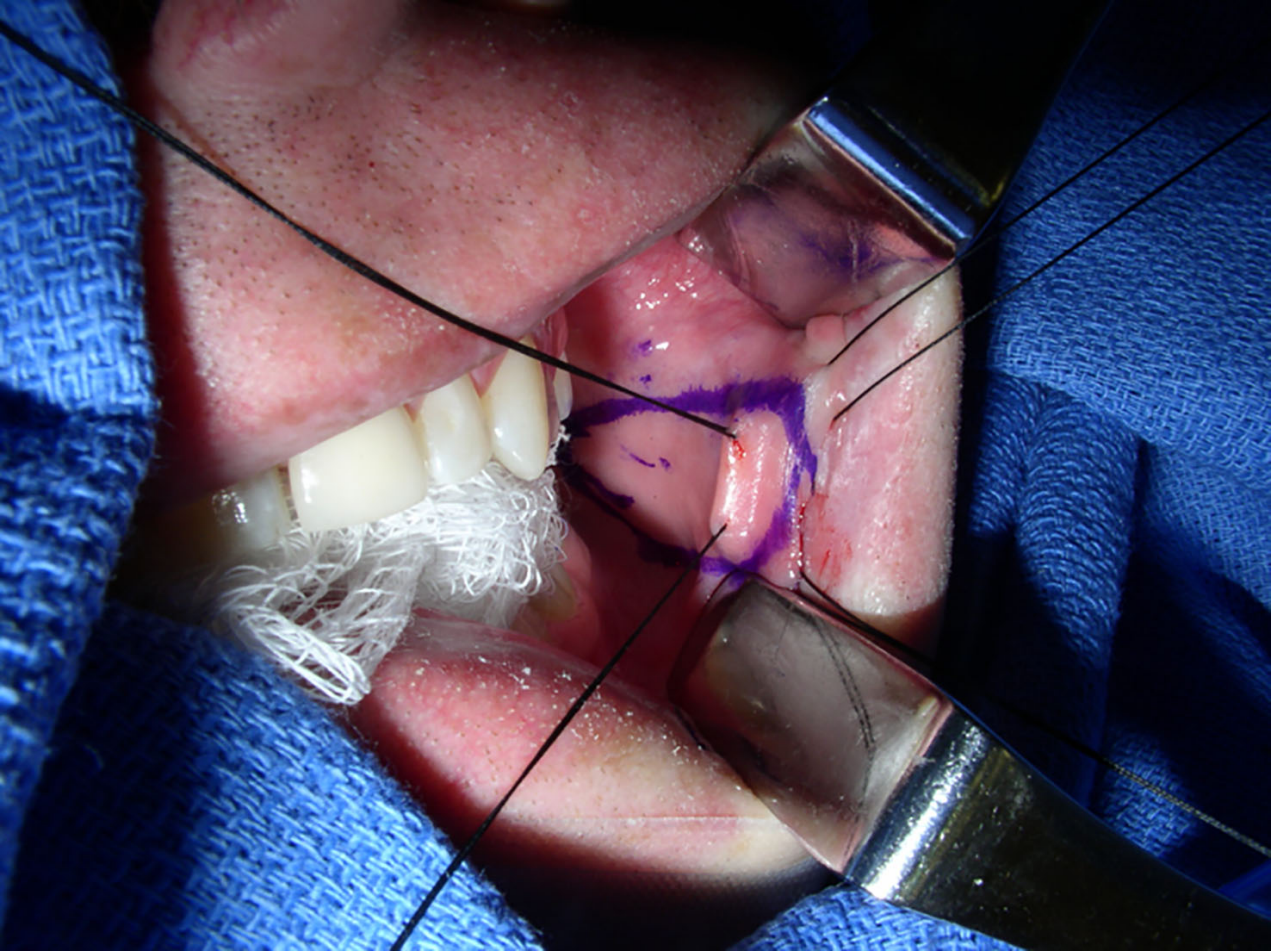
Typical setup for BMG harvest. The dry X-ray-detectable gauze sponge is packed to the contralateral oral space, and three retraction sutures are used with the middle incorporated into the distal apex of the graft. The Army–Navy retractors were used only for photographic exposure of the harvest site and are not typically needed during harvest. The image was obtained intraoperatively at Duke University Medical Center, with the operation completed under routine care. BMG, buccal mucosal graft.

**Figure 2:**
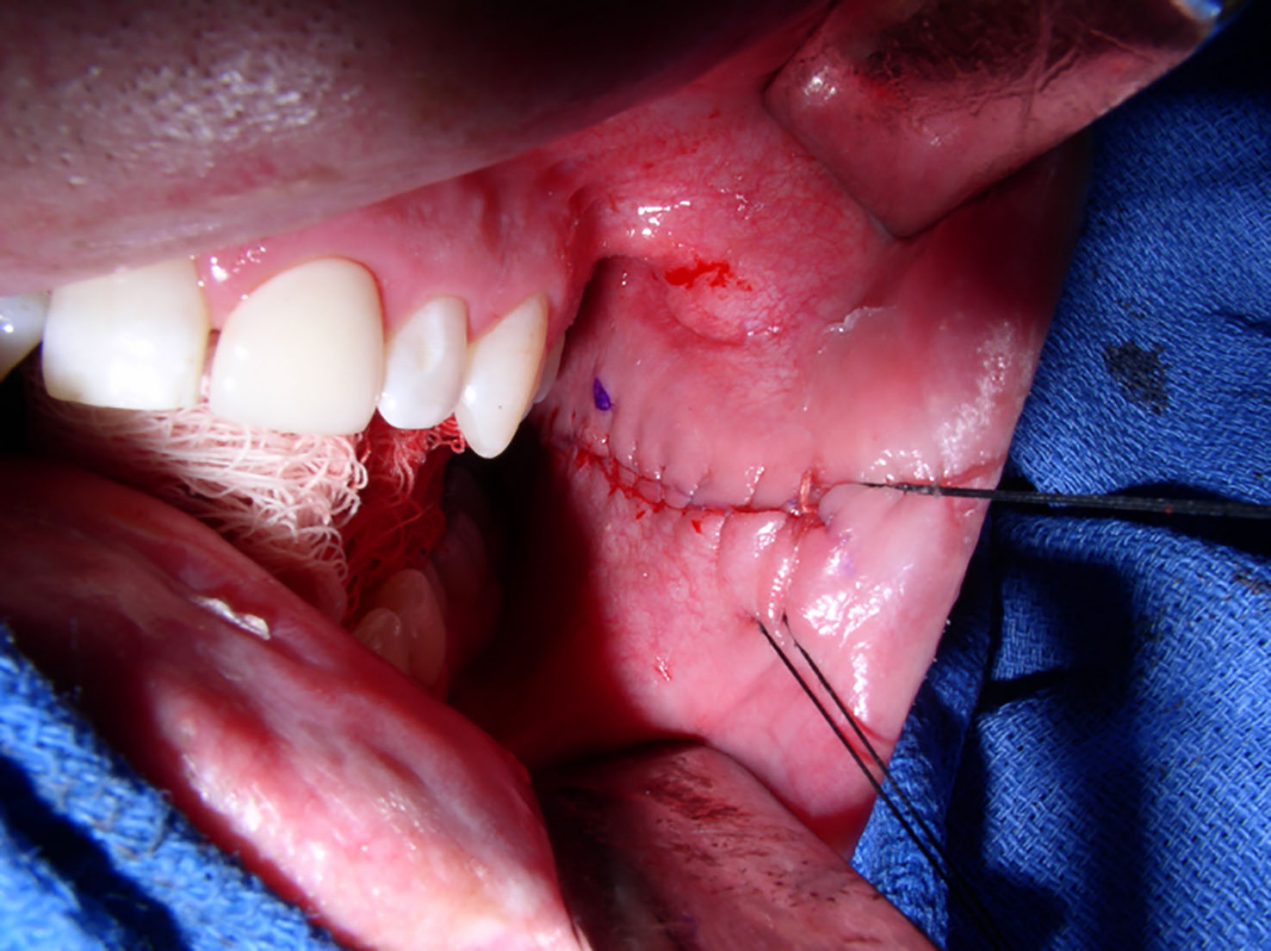
The BMG donor harvest site closed with an absorbable running suture. The blue dot indicates the Stensen’s duct. The image was obtained intraoperatively at Duke University Medical Center, with the operation completed under routine care. BMG, buccal mucosal graft.

At our institution, patients undergoing BMG urethroplasty undergo a standard harvest technique. A separate sterile instrument table is used. Harvest can be completed with a standard endotracheal tube or laryngeal mask airway secured to the contralateral side of harvest. Patients do not receive any oral preparation or antibiotic cleanses preoperatively or intraoperatively. The patient is draped in quartered-off sterile surgical towels. A surgical retractor, such as a Sluder–Jansen mouth retractor, with a tongue blade can be used. Our preference is to simplify the process and use a dry X-ray-detectable gauze sponge and pack the tongue in the contralateral mouth space. This, combined with three robust stay sutures placed 1–2 cm inside the inner vermilion border at the oral commissure and separated at 3–4 cm, is a more than sufficient retraction. Stensen’s duct is marked, and the expected graft length is marked circumferentially in an ellipsoid fashion with the desired width. There should be at least a 3- to 5-mm distance between the graft harvest site and Stensen’s duct. The distal aspect of the graft should be about 1–2 cm from the vermilion border, and the oral commissure retraction stitch can be incorporated into the distal graft apex to allow for further retraction during harvest (**Figure 1**). Using a spinal needle, normal saline is used to hydrodissect the mucosa. The previously marked graft site is incised with a 15 blade and the remainder of the graft is separated from the buccal fat pad and buccinator muscle with sharp scissor dissection. Bovie electrocautery can be used for hemostasis at the graft bed. Our preference is to close the graft site using a running-locking absorbable suture (**Figure 2**). The mouth is then packed with X-ray-detectable gauze sponges soaked in 1% lidocaine with 1:100,000 epinephrine and left until the end of the case. The graft is then de-fatted with scissor dissection down to the white lamina propria, perforated with a 15 blade, and placed in saline.

## Care of the BMG harvest site

Although donor site complications are rare in both historic and contemporary series, oral care pathways and oral antiseptics remain commonly employed. The use of oral antiseptics for BMG urethroplasty is a relatively new development in the urologic literature. In their seminal report in 1992, Dessanti et al. described BMG harvesting as a “septic procedure” with no mention of oral antiseptic use (21). In the first reported adult series utilizing BMGs for urethroplasty the following year, El-Kasaby et al. (1993) reported no mention of oral care regimens or local antiseptic treatments (22). In their report on a two-team technique for buccal harvest in 1996, Morey and McAninch used penicillin G to prevent oral flora infection and made no use of oral antiseptics (6). The use of a povidone-iodine mouth rinse can be first found as a suggestion in the discussion by Burger et al. (1992) for comfort reasons (23), but the practice of using any preoperative oral antiseptics in the urology literature was not described until 2003 (24). Chlorhexidine was probably adopted from infection prevention efforts in other disciplines (24, 25), and was not specifically mentioned in the urologic literature until 2005, by MacDonald and Santucci (26).

Early studies acknowledged that there was no evidence to support the use of aggressive sterilization measures and oral cleanses. Despite this, the use of antibiotics and germicidal mouthwashes in BMG studies was perpetuated with increasing duration and intensity to reduce the potential for infection (**Table 1**).

**Table 1:** Studies of BMG urethroplasty antibiotic regimens and infection rates.

Study (year)	Antibiotic regimen	Postoperative infectious complications
Virasoro et al. (2015) (27)	IV amoxicillin/clavulanic acid and ciprofloxacin 48 hours after surgery, and discharged on ciprofloxacin for 5 days	Five UTIs One case of epididymitis
Vasudeva et al. (2015) (28)	Amoxicillin/clavulanic acid and ceftriaxone started preoperatively and continued for 3 days postoperatively. Levaquin® was administered until catheter removal at 4–5 weeks	Wound infection and graft necrosis in four
Filmore et al. (2014) (29)	No Betadine® given if perioperative antibiotics were given. A small moist oral pack was placed postoperatively	0% donor site infection 4% recipient site infection
Adaqadossi et al. (2013) (30)	Ceftriaxone before surgery and continued for 5 days postoperatively. Povidone-iodine mouthwash started 2 days preoperatively, and it continued 3 days postoperatively	Seven wound infections (three onlay, four inlay) managed by a change in oral antibiotics. No donor site morbidity after 3 months
Pahwa et al. (2013) (31)	Ceftriaxone and amikacin were given before surgery. They were continued for 3 days postoperatively followed by oral antibiotics for another week	Two wound infections managed with IV antibiotics
Hoy NY, Kinnaird A, Rourke KF (2013) (32)	Broad-spectrum antibiotic was given for 48 hours	Six UTIs No donor site infection
Ahmad et al. (2011) (33)	Broad-spectrum antibiotic and metronidazole was given at the time of induction	Seven infections and swelling of the cheek, which settled in 1 week Three superficial wound infections, which responded to antibiotics and sitz baths within 1 week
Francis et al. (2010) (34)	Broad-spectrum antibiotic was given empirically or based on the results of a urine culture	One UTI One case of epididymitis
Arlen et al. (2010) (35)	Antibiotic coverage was for 7 days. Germicidal mouthwash was given QID for 2–3 weeks	One superficial wound infection One abscess/fistula formed

IV, intravenous; UTI, urinary tract infection; QID, four times per day.

Today, oral care regimens and mouthwashes remain commonly employed and a review of the literature demonstrates significant heterogeneity between centers (**Table 2**).

**Table 2:** Variation in BMG harvest oral care pathways.

Study (year)	Preoperatively	Postoperatively
Morán et al.(2019) (36)	None specified	CHX-impregnated gauze packing. External ice pack. CHX rinses TID through to POD7. CLD (cold soups or broths) administered on PODs 1 and 2
Jonnavithula et al. (2019) (37)	CHX BID begins 3 days preoperatively	Allowed to drink orally 6 hours after surgery and advance diet as tolerated. CHX TID through to POD3
Zumrutbas et al. (2019) (38)	CHX started 2–3 days preoperatively	None specified
Soave et al. (2018) (39)	None specified	Daily oral rinsing with chamomile and cooling of the cheek through to POD5
Shalkamy et al. (2017) (40)	Povidone-iodine started 2 days preoperatively	Povidone-iodine mouthwash continued through to POD3
Cakiroglu B, Sinanoglu O, Arda E (2017) (41)	None specified	CLD on POD1, gradually advanced to soft and regular diet in the following days
Joshi et al. (2017) (42)	CHX BID prior to second-stage urethroplasty	None specified
Spilotros et al. (2017) (43)	None specified	Benzydamine hydrochloride-based mouthwash TID for 3 weeks
Barbagli et al. (2016) (44)	CHX BID starting 3 days preoperatively. IV antibiotics started 1 day preoperatively	Ice bag applied to cheek for 24 hours. Cold CLD on POD1. Regular diet POD2. CHX BID for 3 days postoperatively. Oral abx until catheter removal
Lumen et al. (2016) (11)	None specified	Start fluid and food intake POD1. Sodium alginate and potassium hydrogencarbonate oral suspension BID. CHX every morning, evening, and after every meal
Van Putte LV, Win GD (2016) (45)	None specified	Honey-based paste applied to the buccal wound. External ice bag applied to the cheek. Mouth rinsed BID with local antiseptic. On POD1 allowed cold drinks only. On POD2 soft and cold foods are added
Pal et al. (2016) (46)	CHX started 48 hours prior to surgery. Mouth painted and draped. Packing with povidone-iodine-impregnated gauze	Oral pack removed on POD1. No dietary measures specified
Chauhan S, Yadav SS, Tomar V (2016) (47)	None specified	CLD or ice cream on POD1, soft and regular diet gradually introduced in the following days
Vasudeva et al. (2015) (28)	None specified	Oral pack removed and CLD allowed in the evening
Virasoro et al. (2015) (48)	None specified	CHX every 6 hours. Oral intake 12 hours after surgery, and advanced as tolerated
Akyüz et al. (2014) (49)	None specified	Oral mouthwashes containing 0.15 g of benzydamine solution given
Kulkarni et al. (2014) (50))	CHX starting 3 days preoperatively. Abx started 1 day preoperatively	Ice bag is applied on the cheek. CLD with ice cream given on POD1. Regular diet given on POD2. CHX for 3 days postoperatively. Oral abx given until catheter removal
Kaggwa et al. (2014) (51)	CHX mouthwash. Face and cheek prepped with 0.5% CHX intraoperatively	Packing removed in the evening. Mouth rinsed with cold water and diluted mouthwash. Cold oral liquids given on the evening of surgery. POD1–2 semisolid, non-spicy diet given, which was advanced to normal diet as tolerated
Wong et al. (2014) (52)	None specified	CHX after each meal. Normal fluid and solid diet as tolerated
Gimbernat et al. (2014) (53)	None specified	Redon aspiration drainage for 12 hours
Pahwa et al. (2013) (31)	CHX started 2 days prior to surgery	CHX through POD5. Bed rest for 1 week
Aldaqadossi et al. (2013) (30)	Povidone-iodine started 2 days preoperatively	Povidone-iodine continued for 3 days postoperatively
Zimmerman and Santucci (2011) (54)	None specified	Ice applied to the mouth. CHX QID after meals. Advance from CLD to FLD diet on POD1. Regular diet on POD1. Abx until Foley catheter removal (7 days–2 weeks)
Arlen et al. (2010) (35)	None specified	Germicidal mouthwash QID for 2–3 weeks. Soft, mechanical diet given for 2–3 weeks
Sinha et al. (2009) (55)	CHX given in the preoperative period	Oral packing removed in the evening followed by mouth rinse with cold water and diluted mouthwash. Cold CLD started evening of surgery. POD1–2 shift to semisolid, non-spicy diet. Ok for normal diet when patient deems tolerable
Kamp, et al. (2005) (9)	Oral cavity disinfected with iodine-soaked swab. Suprarenine-soaked tampon packing replaced with Scandicaine®-soaked tampon, left *in situ* for 4 hours	The mouth was washed with chamomile tea. There were no diet restrictions

Abx, antibiotics; CHX, chlorhexidine; BID, twice per day; TID, three times per day; CLD, clear liquid diet; POD, postoperative day.

We previously recommended soft food for 48 hours followed by a high-fiber diet, no alcohol for 24 hours, no nuts until the incision was completely healed, and the use of salt water rinses as needed for comfort. Mouthwash regimens were also used, such as Magic Mouthwash (lidocaine, aluminum hydroxide, and magnesium hydroxide), Mouthwash BLM (lidocaine, diphenhydramine, aluminum hydroxide, magnesium hydroxide, and simethicone) or 2% viscous lidocaine solution. Finding no significant benefit, however, we gradually relaxed these measures, and, commensurate with maxillofacial surgical standards, we have never utilized preoperative or intraoperative oral antibiotics. Our current postoperative care pathway includes unrestricted access to food and water, and patients are encouraged to advance their diet as tolerated. We have not found that reducing these measures and simplifying the postoperative pathway results in any deleterious impact on the patient experience.

## Closure of the BMG donor site

There have been several randomized clinical trials (RCTs) and studies evaluating the postoperative complications and morbidity associated with non-closure (NC) compared with closure (C) (**Table 3**).

**Table 3:** Studies evaluating closure and non-closure of BMG harvest site.

Study (year) Type of Study	Non-closure postoperative morbidity	Closure postoperative morbidity
Chua et al. (2019) (56) Systematic review	**Immediate** No difference in pain or oral morbidity **6 months** No difference in pain or oral morbidity Rectangular-shaped BMG NC had lower pain scores (mean difference –0.09, 95% CI –1.7 to –0.10)	
Soave et al. (2018) (39) RCT	**Non-inferior to closure in** **Immediate** Pain: 69% No difference in oral morbidity including mouth opening, numbness, swelling, eating, or smiling **6 months** Pain: 20% No difference in oral morbidity, including mouth opening, numbness, swelling, eating, or smiling	**Immediate** Pain: 52%; *p* = 0.029 **6 months** Pain: 14%; *p* = 0.042
Wong et al (2014) (57) RCT		**Immediate** Improved pain (*p* = 0.08), drinking (*p* = 0.06), and eating (*p* = 0.03) **After 3 weeks** No difference in pain, numbness, tightness, drinking, and eating
Rourke et al. (2012) (58) RCT	**Immediate** Pain: 2.2 Return to diet: 70.8% Full mouth opening: 79.1% Numbness: 62.5% **6 months** Pain: 0.2 Numbness: 4.2%	**Immediate** Pain: 4.1; *p* = 0.07 Return to diet: 19.2%; *p* = 0.01 Full mouth opening: 15.3%; *p* = 0.001 Numbness: 92.3%; *p* = 0.008 **6 months** Pain: 0.3; *p* = 0.63 Numbness: 23.2%; *p* = 0.05
Wood et al. (2004) (59)	5-day postoperative pain score Pain score: 2.26	Pain score: 3.58; *p* < 0.01

RCT, randomized clinical trial.

## Urethroplasty success and outcomes

Recurrence rates are variable for BMG urethroplasty and differ based on urethral stricture location, length of stricture, and etiology. The success of urethroplasty is not universal and is difficult to define (60). A prospective study looking at five ways to define failure included: “1) stricture retreatment, 2) anatomical recurrence on cystoscopy [< 17 fr], 3) peak flow rate < 15 ml/second, 4) weak stream on questionnaire and 5) failure by any of these measures.” (60) The study found that success is highly variable and is inconsistent between definitions (60). This ultimately limits our ability to compare outcomes across studies. A systematic review of BMGs evaluating more than 2,000 urethroplasties noted no difference in dorsal vs. ventral onlay procedures (88.4% and 88.8% at 42.2 and 34.4 months, respectively), lateral onlay (83% at 77 months), the Asopa technique (86.7% at 28.9 months), and the Palminteri technique (90.1% at 21.9 months). (61) **Table 4** includes several studies with definitions of failure and rates of success.

**Table 4:** Rates of urethral stricture recurrence and definitions of recurrence.

Study (year)	Definition of failure	Follow-up month	Rate of success
Levy et al. (2017) (62)	Evaluated age and urethroplasty failure Functional success at 1 year —Any instrumentation Anatomical success at 3 months —Ability to atraumatically pass cystoscope through repair	21.6	Age < 60 years, age > 60 years; *p* = 0.46 86%, 91.4% Age < 60 years, age > 60 years; *p* = 0.21 71.7%, 84%
Lumen et al. (2016) (11)	—Any instrumentation	30	82.8%
Ahyai et al. (2015) (63)	—Isolated post-radiation urethroplasty —Any instrumentation and when *Q*_max_ was < 15 mL/second	26.5	71.1%
Barbagli et al. (2014) (64)	—Any instrumentation	72	Failure-free survival: 78%
Kulkarni et al. (2009) (8)	—Any instrumentation	22	92%
Elliott et al. (2003)	Ventral onlay —Symptom recurrence	47	90%
Morey et al. (1999) (6)	—Any instrumentation	18	100%

Reported complications of BMG substitution urethroplasty are rare or transient. Transient erectile dysfunction has been reported in 26% of patients with BMG substitution, compared with 50% in excision and primary anastomosis, with most recovering at 6 months and 90% of cases completely resolving (65). Even in complex urethral strictures of > 8 cm, the incidence of urethral pseudodiverticulum and penile chordee is reported to be around 3% (66).

## BMG oral harvest complications

Lasting complications associated with BMG harvest are, overall, rare, with many reports noting early transient side effects. Several studies have reported low rates of long-term complications, including pain, oral tightness, numbness, and difficulty with mastication, mouth opening, and speech (18, 58, 59, 67). Pain at the donor site can be a transient side effect after surgery reported postoperatively in 50%–70% of patients in the first week (39). A multivariable analysis from a cohort of 553 patients undergoing BMG harvest reported that 53.2% of patients did not have postoperative pain, 32.4% had slight pain, and rare long-term difficulty with opening the mouth (95.5%), difficulty smiling (98.2%), and dry mouth (95.8%) (68). This study also found a 98.2% patient satisfaction with the procedure, with the only predictive variable for patient dissatisfaction being bilateral graft harvest (68).

## Future directions

Tissue-engineered oral mucosa has been described with the intent to limit the morbidity in patients with long-length urethral strictures or those with recurrences and limited oral mucosa available, such as patients with lichen sclerosis (69, 70). This process involves autologously harvesting oral cells, which are then cultured on epithelial cell sheets, and after 2 weeks the sheets are then tubularized to form a two-layered graft (69). Bhargava et al. utilized tissue-engineered buccal mucosa in five patients with strictures secondary to lichen sclerosis. Buccal mucosal biopsies were taken and propagated using donor de-epithelialized dermis and used for both single- and two-stage procedures. At follow-up, two patients had limited graft take and all patients required further endoscopic treatment (71). Clearly, this is a promising avenue to explore, but further studies are needed before its widespread use.

## Conclusion

BMG remains the gold standard for substitution graft urethroplasty. This review highlights the history of the use and widespread adoption of BMGs, the physiological characteristics of BMG that makes it an ideal graft material, the nuances of harvest and perioperative/preoperative variability in practices, associated complications, and future directions.

## Ethics statement

Written informed consent was obtained from the individual(s) for the publication of any identifiable images or data included in this article.

## Author contributions

JF, AP, and KK contributed to the conception and design of the review; drafting and reviewing of the manuscript; revisions/edits; and a review of references. All authors contributed to the article and approved the submitted version.
